# Association between unintended pregnancy and maternal antenatal care services use in Ethiopia: analysis of Ethiopian demographic and health survey 2016

**DOI:** 10.3389/fmed.2023.1151486

**Published:** 2023-04-19

**Authors:** Ayalnesh Zemene Yalew, Oladapo O. Olayemi, Alemayehu Worku Yalew

**Affiliations:** ^1^Pan African University for Life and Earth Science Institute (Including Agriculture and Health), University of Ibadan, Ibadan, Nigeria; ^2^School of Nursing, St. Paul’s Hospital Millennium Medical College, Addis Ababa, Ethiopia; ^3^Department of Obstetrics and Gynecology, College of Medicine, University of Ibadan, Ibadan, Nigeria; ^4^School of Public Health, College of Health Science, Addis Ababa University, Addis Ababa, Ethiopia

**Keywords:** unintended pregnancy, antenatal care, EDHS, Ethiopia, antenatal care initiation

## Abstract

**Introduction:**

Unintended pregnancy disproportionately affects women in low and middle-income countries including Ethiopia. Previous studies identified the magnitude and negative health outcomes of unintended pregnancy. However, studies that examined the relationship between antenatal care (ANC) utilization and unintended pregnancy are scarce.

**Objective:**

This study aimed to examine the relationship between unintended pregnancy and ANC utilization in Ethiopia.

**Methods:**

This is a cross-sectional study conducted using the fourth and most recent Ethiopian Demographic Health Survey (EDHS) data. The study comprised a weighted sample of 7,271 women with last alive birth and responded to questions on unintended pregnancy and ANC use. The association between unintended pregnancy and ANC uptake was determined using multilevel logistic regression models adjusted for possible confounders. Finally *p* < 5% was considered significant.

**Results:**

Unintended pregnancy accounted for nearly a quarter of all pregnancies (26.5%). After adjusting for confounders, a 33% (AOR: 0.67; 95% CI, 0.57–0.79) lower odds of at least one ANC uptake and a 17% (AOR: 0.83; 95% CI, 0.70–0.99) lower odds of early ANC booking were found among women who had unintended pregnancy compared to women with intended pregnancy. However, this study founds no association (AOR: 0.88; 95% CI, 0.74, 1.04) between unintended pregnancy and four or more ANC visits.

**Conclusion:**

Our study found that having unintended pregnancy was associated with a 17 and 33% reduction in early initiation and use of ANC services, respectively. Policies and programs designed to intervene against barriers to early initiation and use of ANC should consider unintended pregnancy.

## Introduction

Antenatal care service utilization has tremendous benefits for better maternal health outcomes, particularly in low- and middle-income countries (LMIC) where the majority of maternal mortalities are due to preventable causes (1, 2). Studies indicated that ANC service utilization improves maternal and child health outcomes (3, 4). For instance, timely and adequate uptake of ANC reduces neonatal mortality by 34% and decreases the risk of stillbirth, morbidity, and mortality of women and their children (5–7). Moreover, various evidence indicated that ANC utilization significantly reduces adverse pregnancy outcomes (7–13).

According to the study, meeting the sustainable development goal (SDG) targets for maternal and child mortality by 2030 will require 91 percent coverage of at least one ANC and 78 percent coverage of at least four ANC (14). However, only 49.9% and 44.3 percent of women in low- and middle-income countries (LMICs) use at least one ANC and commence it on time, respectively (15).

In 2005, the Government of Ethiopia introduced healthcare reforms to ensure free access to maternity and child services from public institutions. Yet, because of a lack of certain supplies and medications, patients are typically directed to private clinics, incurring out-of-pocket health expenses. In contrast to the fee-free declaration, several government facilities collect user fees for specific services (16–18). This is because the facilities are not compensated for the services provided under the waiver policy, and they are struggling to fund operational expenditures.

According to the 2016 EDHS report, the national coverage of at least one ANC is 62% and only 34% of them have had 4 and above ANC contacts (19), while only 67.3 of women have initiated their first ANC contact in the first Trimester (20). As a result, recognizing the factors that negatively influence ANC service use is critical to improving ANC service and maximizing the advantages of ANC for both mothers and newborns.

Previous evidence showed that none or inadequate use and late initiation of ANC are associated with several socio-demographic and economic (e.g., lower educational level, being a rural resident, and poor wealth index) and obstetric factors (e.g., higher parity) (21–26). However, more research is needed to uncover other characteristics that may affect the appropriate and timely use of ANC.

Unintended pregnancy is a major public health burden in low and middle-income countries (LMICs) because of its association with negative health outcomes, including pregnancy-related complications and maternal and neonatal deaths (27). Likewise, women who have experienced unintended pregnancy are more likely to have intimate partner violence (28), to be socio-economically disadvantaged (29), and to have an increased risk of depression and parenting stress (30). This could be another important mechanism related to non- or inconsistent use and late initiation of ANC among women who have experienced an unintended pregnancy.

Previous studies conducted LMICs including sub-Saharan Africa (SSA) indicate that unintended pregnancy significantly reduces timely initiation and use of ANC (31–34). However, studies conducted in LMICs including Ethiopia to assess the relationship between unintended pregnancy and the three outcomes of ANC (use of at least one ANC, timely initiation of ANC, and adequate number of ANC) were scarce. Moreover, the findings of these few available studies are inconsistent ranging from no association (35–37) to a significant association (32, 34, 38, 39). Varied study locations and designs, small sample sizes, adjustment confounders, and the absence of community-level impacts are all possible explanations for such disparities. Therefore, this study aimed to assess the association between unintended pregnancy and ANC utilization in Ethiopia using the fourth and most recent Ethiopian demographic and health survey data.

## Materials and methods

### Study design and setting

This study is a cross-sectional study conducted in Ethiopia, a developing country whose economy is entirely dependent on agriculture. The entire population of the nation is expected to reach 102.3 million in 2016, with 83% living in rural areas (40). Contraception is the most effective method of avoiding unintended pregnancy, however, only 35% of reproductive-age women who are currently married use modern contraception (19). According to the Ethiopia Demographic and Health Survey (EDHS), the total fertility rate 3 years before the 2016 EDHS was 4.6 children per woman (19).

### Source of data and study population

**Figure 1:**
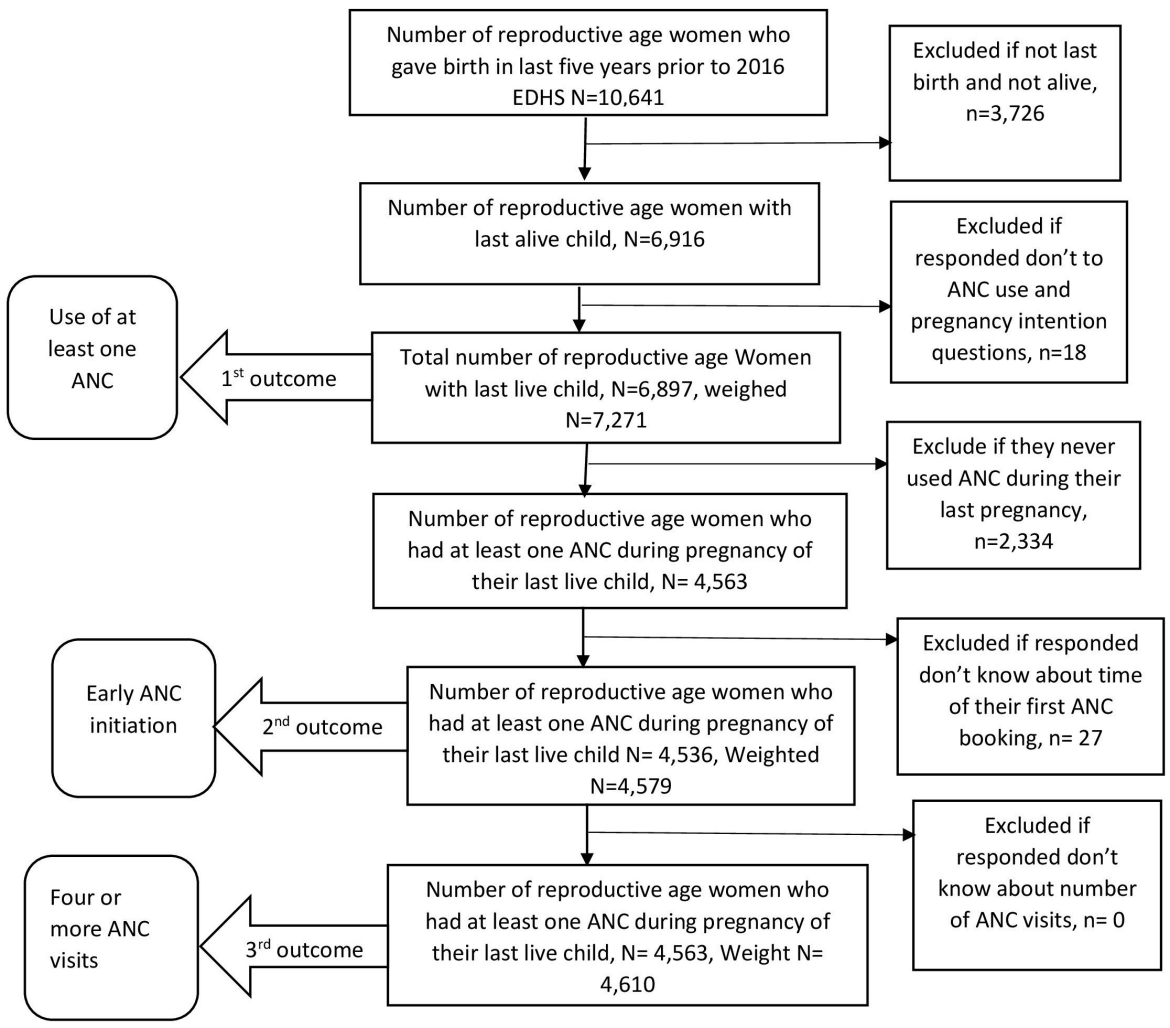
Population flow diagram (weighted number of cases for each outcome).

The Source of data used for analysis was the 2016 Ethiopian Demographic Health Survey (EDHS). It is the most recent nationally representative survey, conducted by the Central Statistical Agency (CSA) from January 18 to June 27, 2016. Samples were taken from all nine regional states as well as two local governments around the country (19). Only women with the last alive birth (the last child who was delivered alive in the last 5 years before the 2016 Ethiopian Demographic and Health Survey) were included in the study. Finally, a total weighted sample of 7,271, 4,579, and 4,610 women was included in the analysis to determine the association between unintended pregnancy and use of at least one ANC, early ANC initiation, and use of four or more ANC visits, respectively. A face-to-face interview was used to collect the data. Detailed inclusion and exclusion procedures are illustrated in Figure 1. For further information, the 2016 EDHS full report is available elsewhere (19).

### Study variables

#### Definition and measurements of outcome variables

In this study, three outcome variables were considered. The first variable was the use of at least one ANC service from a skilled practitioner such as a doctor, midwife, nurse, health officer, or health extension worker (19). It’s divided into two categories: “Yes” and “No.” “Yes” for women who had at least one ANC contact for their most recent live birth, and “No” for those who did not.

The second outcome variable was early ANC initiation. It was grouped into two groups: “Yes” and “No.” Women who started their first ANC within 12 weeks of pregnancy were classed as “Yes” otherwise “No.” The third outcome variable was the number of ANC visits. Women who had four or more ANC visits for their last alive birth were classed as “Yes” while those who had fewer than four visits were classified as “No.” Measurement and definition of the second and the third outcome variable was made based on the WHO 2002 recommendation which was in practice at the time of the 2016 Ethiopian Demographic and Health Survey (41).

#### Predictor variables

Unintended pregnancy was the main predictive variable. In EDHS, pregnancy intendedness was measured using a set of questions that asked women to recall their feelings at the time of conception for each baby born in the previous 5 years. ‘When you were pregnant with [Name of the kid], did you desire the pregnancy then, later, or not at all?’ Women who said their last pregnancy was ‘wanted later’ or ‘not wanted at all’ were classified as having an “unintended” pregnancy, while those who said ‘wanted then’ were classified as having an “intended” pregnancy.

#### Confounding variables

Individual and community-level confounders were taken into account. Age, educational level, marital status, present work, wealth index, media exposure, parity, and religion were included as individual-level variables of the women. The variable household wealth status is a composite measure of a household’s cumulative living standard. It was measured using household assets based on common products such as televisions, bicycles, building materials, domestic animals, land, and other wealth-related qualities. The index was constructed using principal component analysis and categorized into five quintiles poorest, poorer, average, richer, and richest.

The community-level variables included in the analysis were a place of residence, region, and perceived distance to a health institution. Based on women’s exposure to the three media (reading newspapers or magazines, listening to the radio, and watching television); we divided media exposure into three categories: “none.” “infrequent,” and “frequent.” “None” means that the women were not exposed to any of the three media, whilst “infrequent” and “frequent” imply that the women were exposed to the media less than once a week and at least once a week, respectively.

We considered the community-level poverty and literacy to show the overall community-level literacy and poverty. These variables were obtained by aggregating the individual-level wealth status and individual educational level into clusters by using the proportion of those who had individual household wealth and individual educational level. Then community poverty was categorized as “High (<25%),” “medium (25–50%),” “Low (50–75%),” and “Very low (>75%).” Community literacy was also categorized into: “High (<25%),” “moderate (25–50%),” and “Low (>50%).” Additionally, based on geopolitical characteristics and previous literature (42), the region was recategorized into three categories: larger central (Tigray, Amhara, Oromia, and Southern Nations Nationalities and Peoples Region), small peripherals (Afar, Somali, Benishangul, and Gambela), and metropolis (Harari, Dire Dawa, and Addis Ababa).

### Statistical analysis

We applied sampling weights to proceed with the descriptive statistics such as frequency and proportions to adjust for the non-proportional allocation of the sample to strata (urban and rural dwellings) and regions during the survey process. Descriptive analysis using statistical parameters such as Frequencies, percentages, and standard deviation (SD) was then performed.

To examine the relationship between independent and dependent variables, both bivariable and multivariable multilevel logistic regression analyses were conducted. Each variable in the bivariable analysis model was analyzed one by one and those variables with a value of *p* < 0.2 were moved to the multivariable model. Lastly, the adjusted odds ratios (AOR) with a 95% confidence interval were utilized to quantify the measures of association (fixed effects) between the odds of the outcome variables and explanatory variables. A value of p of less than 0.05 was considered statistically significant.

Individual observations with nested structures, such as demographic health survey data, show some connection within a cluster due to similar features. This type of data violates the concept of observational independence, which is required by most statistical analysis techniques (43). As a result, multilevel modeling is better suited to addressing such a problem. This type of modeling allows for precise standard error estimation without conforming to the observation independence hypothesis (43). As a result, for the binary response of the outcome variables, the current study used a two-level mixed-effect binary logistic regression. Intra-class Correlation Coefficient (ICC), which measures the percentage of variance explained by the upper level, was also used to quantify the clustering effect or community variation. The ICC was determined using the formula below (43).


$$ICC=\frac{VA}{VA+\frac{\pi 2}{3}}$$

Where: *V_A_* is community-level variance and $\frac{\pi 2}{3}$ is individual-level variance which is equal to 3.29 (the value of/in case of standard logistic distribution) (43). We built four models based on the premise that community intercepts change but coefficients remain constant. The first is the Null model, which is a model with no explanatory variables. The second model is based solely on individual-level variables. Model III is a community-level variable model. The fourth model is a composite model that takes individual and community-level factors into account.

A null model was fitted to each outcome variable analysis to support the requirement for multilevel analysis and to evaluate the clustering impact or between community variations by evaluating ICC. The value of ICC was determined to be high, which validates the use of a multilevel model.

To measure the level of contribution of individual and community-level variables in explaining the study’s outcome variables, the proportional change in variance (PCV) was calculated using the null model as a reference. It was calculated using the formula: $PCV=\frac{\mathrm{Vo}-\mathrm{Vi}}{\mathrm{Vo}}$ (43). Where: *Vo* is variance in the null model and *Vi* is variance in the consecutive models.

Moreover, to differentiate the best model fit, Akaike Information Criteria (AIC) was calculated. A model with the lowest AIC value was considered the best-fit model (43).

## Results

### Sociodemographic and reproductive characteristics of the study participants

A total of 7,271 women who gave birth in the 5 years before the 2016 EDHS and responded to questions about ANC usage and pregnancy intentions regarding their last alive birth were included in the analysis. The mean age of the participants was 29.56 (SD = 6.89) years. Six in ten (62.9%) of the women had no education. It was found that 87.06% of women were from rural areas (Table 1). Of the total participants around 26.49% had reported unintended pregnancy. Approximately 36.6% of women stated they did not receive at least one professional ANC visit during their most recent pregnancy. Furthermore, 67.5% of women had initiated their first ANC lately (after 12 weeks of pregnancy), and 49.0% had inadequate (<4) ANC visits.

**Table 1:** Selected sociodemographic and reproductive characteristics of the study participants, EDHS 2016 (weighted sample *N* = 7,271).

Variables		Frequency	Percentage
Maternal age	19–24	1,733	23.8
	25–34	3,681	50.6
	35–49	1,857	25.5
Maternal education	No education	4,573	62.9
	Primary	2,068	28.4
	Secondary and above	630	6.1
Marital status	Married	6,827	93.9
	Unmarried	444	26.5
Pregnancy intention	Intended	5,345	73.5
	Unintended	1,926	26.5
Use of at least 1 ANC	Yes	4,610	63.4
	No	2,661	36.6
Early ANC initiation (*N* = 4,579)	Yes (before 12 weeks)	1,488	32.5
	No (after 12 weeks)	3,091	67.5
4 or more ANC visits (*N* = 4,610)	No (<4 visits)	2,259	49.0
	Yes (4 or more visits)	2,351	51.0
Parity	Prim gravida	1,393	19.1
	Multipara	3,880	53.4
	Grand multipara	1,998	27.5
Religion	Orthodox	7,279	38.2
	Catholic	66	0.9
	Protestant	1,594	21.9
	Muslim	2,673	36.8
	Other *	159	2.2
Residence	Rural	6,330	87.1
	Urban	941	12.9
Region	Large central	6,609	90.9
	Small peripheral	419	5.8
	Metropolitan	243	3.3
Media exposure	None	4,781	65.8
	Infrequent	1,071	14.7
	Frequent	1,419	19.5
Household wealth status	Poorest	1,581	21.7
	Poorer	1,592	21.9
	Average	1,519	20.9
	Richest	1,355	18.6
	Richer	1,224	16.8
Distance to a health facility	Big problem	4,230	58.2
	Not big problem	3,041	41.8

* Other religion includes traditional and other.

**Figure 2:**
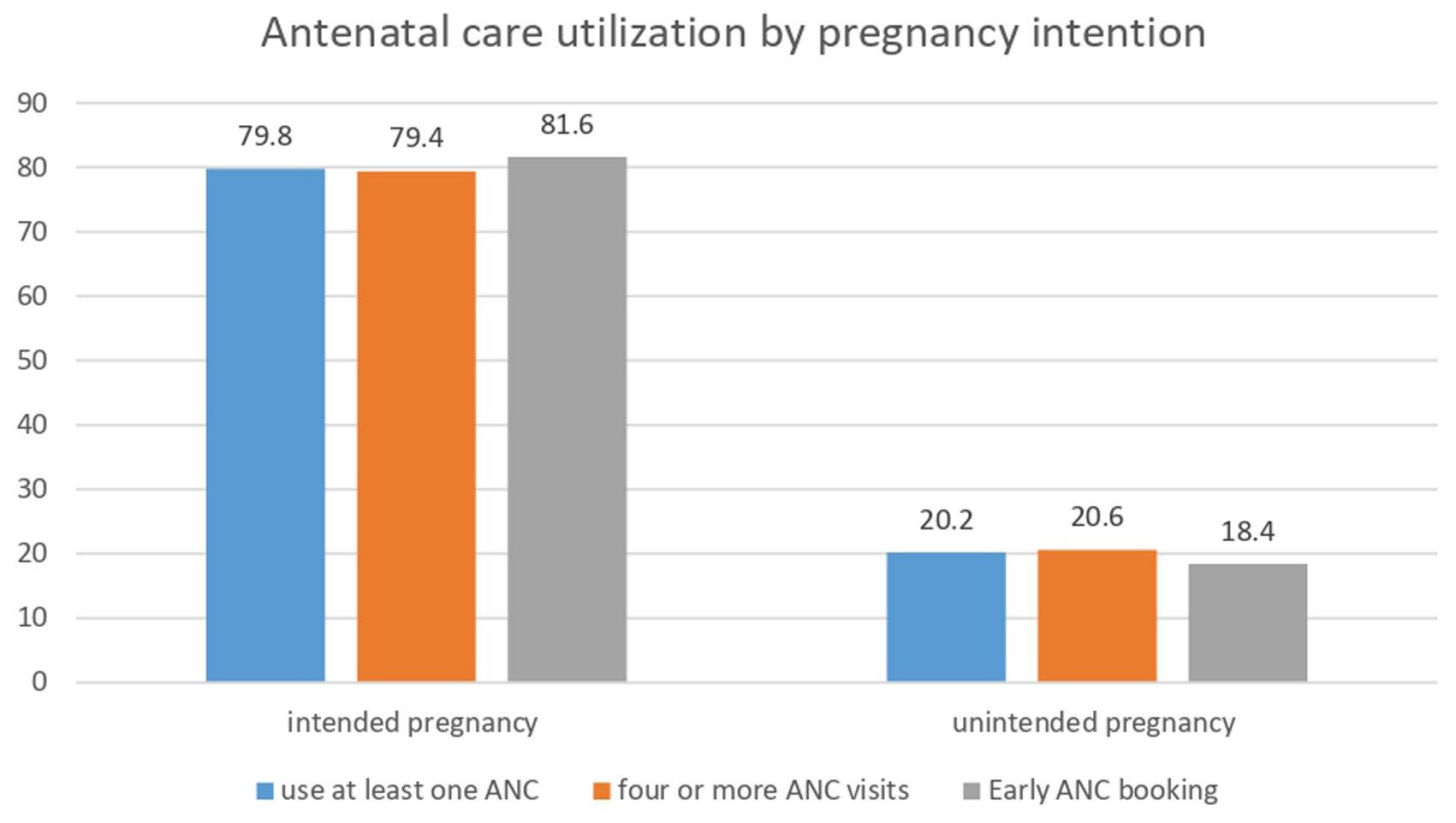
Antenatal care service utilization across pregnancy intention in Ethiopia, EDHS 2016.

There was significant variation in ANC service utilization across pregnancy intentions. A small proportion (20.2%) of women with unintended pregnancy utilized at least one ANC during the pregnancy of their last child while a substantial proportion (79.6%) of women with intended pregnancy used at least one ANC. Likewise, only 18.4% of women with unintended pregnancy booked their ANC early while 81.6% of women with intended pregnancy booked their ANC early (Figure 2).

The four models (model I, II, III, and IV) were separately conducted to determine the association between unintended pregnancy and at least one ANC service use, early initiation of first ANC, and use of four or more ANC visits (Supplementary Tables S1–S3). The Akaike information criterion (AIC), Bayesian information criterion (BIC), and proportion change in variance (PCV) were compared in each model to determine which the best was. The model with the lowest AIC and BIC values as well as the highest PCV was chosen. Model IV (mixed model) was chosen as the best model based on these criteria (Supplementary Tables S1–S3).

Table 2 shows the odds ratios of the three outcome variables produced in the final model (model IV), while (Supplementary Tables S1–S3) show all model results. The odds of using at least one ANC service and early first ANC booking were 33% (AOR: 0.67; 95% CI, 0.57, 0.79) and 17% (AOR: 0.83; 95% CI, 0.70, 0.99) lower among women who had unintended pregnancy than women who had intended pregnancy, respectively (Table 2). Surprisingly, this study revealed no relationship between unintended pregnancy and the number of ANC visits (AOR: 0.88; 95% CI, 0.74, 1.04) (Table 2).

**Table 2:** Multilevel modeling to determine the relationship between unintended pregnancy and use of at least one ANC, use of four or more ANC visits, and early ANC booking in Ethiopia using 2016 EDHS.

Variables		At least one ANC	Four or more ANC	Early ANC booking
		AOR (95% CI)	AOR (95% CI)	AOR (95% CI)
Pregnancy intention	Intended	Ref	Ref	Ref
	Unintended	0.67 (0.57, 0.79) ***	0.88 (0.74, 1.04)	0.83 (0.70, 0.99) *
Maternal age	19–24	Ref	Ref	Ref
	25–34	1.12 (0.92, 1.35)	1.24 (1.02, 1.50)	1.02 (0.85, 1.22)
	35–49	0.81 (0.63, 1.05)	1.28 (0.99, 1.66)	0.06 (0.82, 1.37)
Maternal education	No education	Ref	Ref	Ref
	Primary	1.77 (1.48, 2.11) ***	1.21 (1.02, 1.44)	1.12 (0.94, 1.34)
	Secondary and above	2.70 (1.89, 3.86) ***	1.78 (1.38, 2.31)	1.45 (1.15, 1.85)**
Currently marital status	Unmarried	Ref	Ref	Ref
	Married	1.51 (1.15, 1.98)**	0.82 (0.63, 1.08)	0.88 (0.68, 1.14)
Religion	Orthodox	Ref	Ref	Ref
	Catholic	0.36 (0.15, 0.83)*	1.12 (0.45, 2.79)	1.67 (0.71, 3.92)
	Protestant	0.53 (0.40, 0.69)***	0.72 (0.56, 0.92)	0.77 (0.62, 0.97)*
	Muslim	0.74 (0.57, 0.96)***	0.65 (0.52, 0.79)	0.92 (0.76, 1.12)
	Other	0.29 (0.17, 0.54)***	0.97 (0.43, 2.17)	1.06 (0.49, 2.32)
Having current work	No	Ref	Ref	Ref
	Yes	1.17 (0.99, 1.36)	1.01 (0.87, 1.18)	1.01 (0.87, 1.18)
Parity	Primiparous	Ref	Ref	Ref
	Multiparous	0.76 (0.61, 0.95)*	0.89 (0.73, 1.09)	0.82 (0.68, 0.99)*
	Grand multiparous	0.68 (0.51, 0.89)**	0.82 (0.62, 1.09)	0.58 (0.44, 0.76)***
Health insurance cover	No	Ref	Ref	Ref
	Yes	1.43 (0.91, 2.24)	1.44 (0.99, 2.07)	1.25 (0.90, 1.74)
Wealth index	Poorest	Ref	Ref	Ref
	Poorer	1.30 (1.06, 1.59)*	1.27 (1.01, 1.59)	0.97 (0.77, 1.24)
	Average	1.42 (1.12, 1.79)**	1.19 (0.92, 1.54)	1.21 (0.93, 1.57)
	Richest	1.51 (1.16, 1.96)**	1.44 (1.10, 1.90)	1.08 (0.81, 1.43)
	Richer	1.72 (1.19, 2.47)**	1.67 (1.19, 2.34)	1.03 (0.74, 1.45)
Media exposure	None	Ref	Ref	Ref
	Infrequent	1.45 (1.17, 1.80)**	1.04 (0.85, 1.28)	1.12 (0.91, 1.37)
	Frequent	1.77 (1.40, 2.23)***	1.31 (1.07, 1.61)	0.99 (0.81, 1.21)
Community-level factors				
Residence	Rural	Ref	Ref	Ref
	Urban	1.99 (1.27, 3.09)**	1.02 (0.73, 1.43)	1.57 (1.16, 2.12)**
Region	Large central	Ref	Ref	Ref
	Small peripheral	0.73 (0.55, 0.98)**	1.04 (0.81, 1.32)	1.06 (0.85, 1.31)
	Metropolitan	1.56 (1.02, 2.37)*	1.74 (1.29, 2.34)	2.84 (2.22, 3.62)***
Distance to health facility	Big problem		Ref	Ref
	Not big problem	1.45 (1.24, 1.68)***	0.97 (0.84, 1.14)	0.96 (0.82, 1.11)
Community literacy	High	Ref	Ref	Ref
	Moderate	1.59 (1.15, 2.22)**	1.04 (0.76, 1.41)	1.31 (0.98, 1.73)
	Low	2.92 (2.00, 4.27)***	1.51 (1.07, 2.12)	1.15 (0.84, 1.58)
Community poverty	High	Ref	Ref	Ref
	Medium	1.01 (0.71, 1.45)	0.94 (0.70, 1.26)	0.81 (0.62, 1.06)
	Low	1.59 (1.06, 2.38)*	0.90 (0.64, 1.25)	0.83 (0.61, 1.13)
	Very low	0.73 (0.49, 1.10)	0.69 (0.48, 0.99)	1.11 (0.79, 1.54)
Random effect and model fitness				
Random effect	Community variance (SE)	0.75 (0.210)	0.50 (0.080)	0.26 (0.058)
	ICC (%)	24.01	13.28	7.37
	PCV (%)	75.73	53.27	65.79
Model fitness	Log likelihood	−3305.29	−2814.66	−2779.70
	AIC	6672.59	5691.32	5621.41
	BIC	6884.59	5890.52	5820.54

*Significant at value of *p* < 0.05; **significant at value of *p* < 0.01; ***significant at value of *p* < 0.001. AOR (95%CI), Adjusted Odds Ratio at 95% confidence level; AIC, Akaike information criterion; BIC, Bayesian information criterion; PCV, proportion change in variance; ICC, Intraclass correlation.

After controlling for several confounders, maternal educational status, marital status, religion, level of media exposure, residence, region, perceived distance to the health facility, parity, and community literacy was significantly associated with the use of at least one ANC service (Table 2). Likewise, maternal educational status, parity, and residence were associated with early initiation of ANC service (Table 2).

## Discussion

Using recent and nationally representative Ethiopian Demographic and Health Survey data, the main goal of this study was to determine whether or not unintended pregnancy has an association with at least one ANC uptake, early first ANC booking, and four or more ANC use. More than a quarter of women (26.5%) who had a live birth 5 years before the survey said their most recent pregnancy was unintended at the time of conception. The proportion of unintended pregnancy in this study is lower than the proportion in previous studies (44–46). This might be attributed to social desirability bias owing to the subject’s socially sensitive character, as well as recall bias due to retrospective data collection.

Women who reported unintended pregnancies had 33 and 17% lower odds of at least one ANC uptake and an early first ANC booking, respectively, when compared to women who reported intended pregnancy. This indicates the risk of negative maternal and neonatal outcomes will be increased following the lack of at least one ANC or late ANC initiation of ANC. As a result, new strategies are needed to improve ANC uptake of women with unintended pregnancies through early detection of women who are pregnant unintentionally and providing a link to ANC service at a local health facility.

The risk of maternal and perinatal problems is lowered when women receive at least one ANC from a qualified care provider. It drops the risk of neonatal death and stillbirth by 58 and 66%, respectively, (47). In LMICs, the SDGs call for universal health coverage and a reduction in maternal and child deaths that could be prevented (48). According to current evidence, 91 percent of at least one ANC and 78% of four or more ANC uptake is required to meet SDG targets. However, current ANC coverage in low- and middle-income countries falls far from the required coverage, with only 49.9% and 44.3 percent of women having used at least one ANC and early ANC booking, respectively (15).

Our study revealed a negative relationship between unintended pregnancy and the uptake of at least one ANC, which will make it difficult for Ethiopia to meet its SDG targets. This study was consistent with studies conducted in LMICs including Ethiopia (31, 33, 49, 50). This could be because women who have unintended pregnancies are less financially and emotionally prepared than women who have planned pregnancies (51). Furthermore, women who have unwanted pregnancies are at risk of sexual violence and they may not have the support of their spouses or partners (52).

This study revealed that the first ANC booking before 12 weeks is substantially associated with unintended pregnancy. This conclusion was supported by previous research in Rwanda (53), Tanzania (54), and Bangladesh (49). Women may delay their first ANC booking for a variety of reasons, including delayed recognition of the possibility of unintended pregnancy, fear of others noticing unintended pregnancy or hoping for a spontaneous termination of pregnancy, being unhappy about the occurrence of pregnancy, and lack of financial and emotional preparation for their health and that of the growing fetus (55–57).

Unlike previous studies (33, 34, 49) this study found no association between unintended pregnancy and four or more ANC visits. This could be due to methodological (sample size) and sociodemographic differences. However, a strong association has been observed between four or more ANC visits and late ANC initiation. Women who had late ANC bookings were 72% less likely to have four or more ANC visits. This indicates early ANC booking is necessary not only for early detection of pregnancy-related complications but also to attend the recommended number of ANC visits (15).

Several individual and community-level factors including Women’s primary, secondary, and higher education, married marital status, wealthiest wealth status, frequent media exposure, urban residence, metropolitan region, no big problem with distance to the health facility, and high community literacy were also found to be associated with increased likelihood of using at least one ANC, whereas multiparty, grand multiparty, catholic, and protestant religion was found to be associated with lower odds of using at least one ANC. These findings were in line with previous studies conducted in Ethiopia (58, 59) and China (60).

The study’s main strength is that we analyzed nationally representative data in compliance with WHO recommendations for ANC initiation and the minimum number of ANC visits required at the time of the survey. Furthermore, the findings of this study are more credible because of the appropriate statistical adjustments for the survey design and modeling for confounding effects.

Despite its strengths, the research has certain weaknesses. Because of the retrospective reporting of the timing of the first ANC booking and the number of ANC visits, there may be a recall bias. Furthermore, due to social desirability concerns, unintended pregnancy may be underreported. Furthermore, this study used data from the 2016 EDHS, which may be considered outdated; However, as per our extensive search and also active involvement in the Ethiopian healthcare system, no major changes to maternal healthcare policy or programs have occurred since the 2016 data collection. We have not yet seen a special or new intervention that will improve antenatal care service use, particularly among women who are at risk for unintended pregnancy. Therefore, though the data is dated (collected in 2016), we strongly believe that the recommendation of this study is still valid to develop specific interventions to improve antenatal care service. Moreover, no other nationally representative survey was conducted after the 2016 EDHS except the 2019 mini-survey which lacks data on the main variables (pregnancy intention) of the study.

## Conclusion

In Ethiopia, around 26.5% of most recent live births were unintended at the time of conception. A significant variation of at least one ANC uptake and early ANC initiation was observed across pregnancy intention. Women with unintended pregnancy have had a lower percentage of at least one ANC uptake and a higher percentage of late ANC initiation. When comparing women with unintended pregnancies to women with intended pregnancies, the chances of using at least one ANC and initiating ANC early were 33 and 17% lower, respectively. However, unintended pregnancy was not found to be associated with four or more ANC visits.

This is a good implication that apart from individual and community level factors, unintended pregnancy challenges Ethiopia’s ability to meet its SDG targets of universal health coverage and reduce preventable maternal and newborn deaths by lowering the likelihood of using at least one ANC and increasing the likelihood of late ANC initiation. Policymakers, program administrators, and clinicians striving to achieve the SDGs must consider unintended pregnancy while working to address various levels of barriers to ANC usage and early ANC scheduling. We further recommend researchers conduct qualitative studies which further explore reasons for not using ANC services among women with unintended pregnancy.

## Data availability statement

The raw data supporting the conclusions of this article will be made available by the authors, without undue reservation.

## Ethics statement

The studies involving human participants were reviewed and approved by the Institute for Advanced Medical Research and Training (IAMRAT), College of Medicine, University of Ibadan, Ibadan, Nigeria (ref NHREC/05/01/2008a). The patients/participants provided their written informed consent to participate in this study.

## Author contributions

All authors equally contributed to the conceptualization, data extraction, analysis, interpretation, fund acquisition, validation, visualization, original draft writing, editing of the manuscript, read, and approved the final manuscript and the journal to which it was submitted.

## Funding

This work was supported by the African Union Commission (AU), Addis Ababa, Ethiopia through the Pan African University Life and Earth Science Institute (PAULESI), University of Ibadan, Nigeria.

## Conflict of interest

The authors declare that the research was conducted in the absence of any commercial or financial relationships that could be construed as a potential conflict of interest.

## Publisher’s note

All claims expressed in this article are solely those of the authors and do not necessarily represent those of their affiliated organizations, or those of the publisher, the editors and the reviewers. Any product that may be evaluated in this article, or claim that may be made by its manufacturer, is not guaranteed or endorsed by the publisher.
