# Supplementary material for: Association between unintended pregnancy and maternal antenatal care services use in Ethiopia: analysis of Ethiopian demographic and health survey 2016
Source: Front Med (Lausanne). 2023 Apr 19;10:1151486. doi: 10.3389/fmed.2023.1151486 (PMC10155231; doi:10.3389/fmed.2023.1151486)
Supplement: Supplementary file 1 [file Data_Sheet_1.pdf]

Table 3 Association between unintended pregnancy and at least one ANC uptake in Ethiopia, Multilevel analysis using EDHS 2016.

**Variables**		Model I (Empty model)	Model II AOR(95% CI)	Model III AOR (95% CI)	Model IV AOR (95% CI)
Unintended pregnancy	No		Ref		Ref
	Yes		0.71 (0.60, 0.84)***		0.67 (0.57, 0.79) ***
**Other Individual-level factors**					
Maternal age	19-24		Ref		Ref
	25-34		1.20 (0.99, 1.46)		1.12 (0.92, 1.35)
	35-49		0.93 (0.73, 1.20)		0.81 (0.63, 1.05)
Maternal education	No education		Ref		Ref
	Primary		2.02 (1.69, 2.40)*		1.77 (1.48, 2.11)***
	Secondary & above		3.49 (2.46, 4.96)***		2.70 (1.89, 3.86)***
Marital status	Not married		Ref		Ref
	married		1.43 (1.09, 1.87)**		1.51 (1.15, 1.98)**
Religion	Orthodox		Ref		
	Catholic		0.34 (0.15, 0.80)*		0.36 (0.15, 0.83)*
	Protestant		0.50 (0.38, 0.66)***		0.53 (0.40, 0.69)***
	Muslim		0.58 (0.46, 0.74)***		0.74 (0.57, 0.96)***
	Other		0.26 (0.14, 0.47)***		0.29 (0.17, 0.54)***
Having current work	No		Ref		Ref
	Yes		1.21 (1.03, 1.41)		1.17 (0.99, 1.36)
Parity	Primiparous		Ref		Ref
	Multiparous		0.74 (0.59, 0.92)**		0.76 (0.61, 0.95)*
	Grand multiparous		0.62 (0.47, 0.82)**		0.68 (0.51, 0.89)**
Health insurance cover	No		Ref		Ref
	Yes		1.45 (0.93, 2.28)		1.43 (0.91, 2.24)
Wealth index	poorest		Ref		Ref
	Poorer		1.65 (1.35, 2.00)***		1.30 (1.06, 1.59)*
	Average		1.92 (1.54, 2.39)***		1.42 (1.12, 1.79)**
	Richest		2.17 (1.69, 2.76)***		1.51 (1.16, 1.96)**
	Richer		4.79 (3.54, 6.50)***		1.72 (1.19, 2.47)**
Media exposure	None		Ref		Ref
	Infrequent		1.49 (1.20, 1.85)***		1.45 (1.17, 1.80)**
	Frequent		1.91 (1.52, 2.41)***		1.77 (1.40, 2.23)***
**Community-level factors**					
Residence	Rural			Ref	Ref

	Urban			3.42 (2.28, 5.14)***	1.99 (1.27, 3.09)**
Region	Large central			Ref	Ref
	Small peripheral			0.65 (0.49, 0.85)**	0.73 (0.55, 0.98)**
	Metropolitan			1.81 (1.22, 2.71)*	1.56 (1.02, 2.37)*
Distance to Health facility	Big problem			Ref	
	Not big problem			1.54 (1.33, 1.78)***	1.45 (1.24, 1.68)***
Community literacy	Low			Ref	Ref
	Moderate			1.90 (1.37, 2.65)***	1.59 (1.15, 2.22)**
	High			4.11 (2.84, 5.92)***	2.92 (2.00, 4.27)***
Community poverty	Low			Ref	Ref
	Medium			0.81 (0.57, 1.16)	1.01 (0.71, 1.45)
	High			1.21 (0.81, 1.81)	1.59 (1.06, 2.38)*
	Very high			0.50 (0.34, 0.74)**	0.73 (0.49, 1.10)
Random effect & model fitness					
Random effect	Community variance (SE)	3.09 (0.289)	1.27(0.240)	1.08(0.228)	0.75(0.210)
	ICC (%)	48.5	27.88	25.85	24.01
	PCV (%)	Ref	58.89	65.05	75.73
Model fitness	Log likelihood	-3691.86	-3382.10	-456.0623	-3305.29
	AIC	7387.72	6808.21	6934.13	6672.59
	BIC	7401.39	6958.66	7009.35	6884.59

*significant at p<0.05; **significant at p<0.01; ***significant at p<0.001, AOR (95%CI): Adjusted Odds Ratio at 95% confidence level, AIC: Akaike information criterion, BIC: Bayesian information criterion, PCV: proportion change in variance, ICC: Intraclass correlation

Table 4 Association Between Unintended Pregnancy and Early ANC Initiation in Ethiopia, EDHS 2016.

**Variables**		Model I (Empty model)	Model II AOR(95% CI)	Model III AOR (95% CI)	Model IV AOR (95% CI)
Unintended pregnancy	No		Ref		Ref
	Yes		0.86(0.72, 1.02)		0.83(0.70, 0.99) *
**Other Individual factors**					
Maternal age	19-24		Ref		Ref
	25-34		1.11(0.92, 1.33)		1.02(0.85, 1.22)
	35-49		1.22(0.94, 1.57)		0.06(0.82, 1.37)
Maternal education	No education		Ref		Ref
	Primary		1.19(1.00, 1.42)*		1.12(0.94, 1.34)
	Secondary+		1.64(1.30, 2.07)***		1.45(1.15, 1.85)**
Marital status	Married		Ref		Ref
	Unmarried		0.84(0.65, 1.08)		0.88(0.68, 1.14)
Religion	Orthodox		Ref		Ref
	Catholic		1.58(0.66, 3.73)		1.67(0.71, 3.92)
	Protestant		0.72(0.58, 0.91)**		0.77(0.62, 0.97)*
	Muslim		1.07(0.89, 1.28)		0.92(0.76, 1.12)
	Other		0.94(0.42, 2.10)		1.06(0.49, 2.32)
Having work	No		Ref		Ref
	Yes		1.01(0.87, 1.17)		1.01(0.87, 1.18)
Parity	Primiparous (1)		Ref		Ref
	Multipara (2-5)		0.78(0.65, 0.94)*		0.82(0.68, 0.99)*
	Grand multipara (6+)		0.51(0.39, 0.68)***		0.58(0.44, 0.76)***
Health insurance cover	No		Ref		Ref
	Yes		1.21(0.87, 1.69)		1.25(0.90, 1.74)
Wealth index	poorest		Ref		Ref
	Poorer		0.94(0.75, 1.19)		0.97(0.77, 1.24)
	Average		1.16(0.91, 1.47)		1.21(0.93, 1.57)
	Richest		1.05(0.82, 1.36)		1.08(0.81, 1.43)
	Richer		1.86(1.44, 2.39)***		1.03(0.74, 1.45)
Media exposure	None		Ref		Ref
	Infrequent		1.13(0.92, 1.39)		1.12(0.91, 1.37)
	Frequent		1.01(0.90, 1.35)		0.99(0.81, 1.21)
**Community-level factors**					

Residence	Rural			Ref	Ref
	Urban			1.78(1.39, 2.29)***	1.57(1.16, 2.12)**
Region	Large central			Ref	Ref
	Small peripheral			0.99(0.81, 1.21)	1.06(0.85, 1.31)
	Metropolitan			2.83(2.24, 3.56)***	2.84(2.22, 3.62)***
Distance to Health facility	Big problem			Ref	Ref
	Not big problem			1.00(0.86, 1.16)	0.96(0.82, 1.11)
Community literacy	Low			Ref	Ref
	Moderate			1.36(1.03, 1.81)*	1.31(0.98, 1.73)
	High			1.28(0.96, 1.72)	1.15(0.84, 1.58)
Community poverty	Low			Ref	Ref
	Moderate			0.78(0.60, 1.01)	0.81(0.62, 1.06)
	High			0.79(0.59, 1.05)	0.83(0.61, 1.13)
	Very high			1.01(0.75, 1.35)	1.11(0.79, 1.54)
Random effect & model fitness					
Random effect	Community variance (SE)	0.76(0.98)	0.57(0.099)	0.34(0.059)	0.26(0.058)
	ICC (%)	18.83	11.13	7.97	7.37
	PCV (%)	Ref	25.0	55.26	65.79
Model fitness	Log likelihood	-2935.83	-2837.93	-2813.61	-2779.70
	AIC	5875.66	5719.85	5649.22	5621.41
	BIC	5888.51	5861.17	5719.88	5820.54

*significant at p<0.05; **significant at p 0.01; ***significant at p<0.001, AOR (95%CI): Adjusted Odds Ratio at 95% confidence level, AIC: Akaike information criterion, BIC: Bayesian information criterion, PCV: proportion change in variance, ICC: Intraclass correlation

Table 5:Association Between Unintended Pregnancy and Use of Four or more ANC Visits in Ethiopia, 2016 EDHS.

**Variables**	Model I (empty model)	Model II AOR (95% CI)	Model II AOR (95% CI)	Model IV AOR (95% CI)
**Unintended pregnancy**				
No		ref		Ref
Yes		0.90(0.76, 1.07)		0.88(0.74, 1.04)
**Individual factors**				
**Maternal age**				
19-24		ref		Ref
25-34		1.31(1.08, 1.58)**		1.24(1.02, 1.50)*
35-49		1.41(1.09, 1.83)**		1.28(0.99, 1.66)
**Maternal education**				
No education		Ref		Ref
Primary		1.32(1.12, 1.57)**		1.21(1.02, 1.44)*
Secondary+		1.99(1.56, 2.57)***		1.78(1.38, 2.31)***
**Marital status**				
Married		Ref		Ref
Unmarried		0.80(0.61, 1.05)		0.82(0.63, 1.08)
**Religion**				
Orthodox		Ref		Ref
Catholic		1.13(0.45, 2.82)		1.12(0.45, 2.79)
Protestant		0.74(0.58, 0.93)*		0.72(0.56, 0.92)**
Muslim		0.65(0.54, 0.79)***		0.65(0.52, 0.79)***
Other		0.90(0.40, 2.02)		0.97(0.43, 2.17)
**Current work**				
No		Ref		Ref
Yes		1.02(0.87, 1.19)		1.01(0.87, 1.18)
**Parity**				
Primiparous (1)		Ref		Ref
Multipara (2-5)		0.87(0.71, 1.06)		0.89(0.73, 1.09)
Grand multipara (6+)		0.75(0.57, 0.99)		0.82(0.62, 1.09)
**Health insurance cover**				
No		Ref		Ref
Yes		1.39(0.97, 1.99)		1.44(0.99, 2.07)
**Household Wealth status**				
poorest		Ref		Ref
Poorer		1.40(1.12, 1.75)**		1.27(1.01, 1.59)*
Average		1.39(1.10, 1.75)**		1.19(0.92, 1.54)
Richest		1.75(1.37, 2.23)***		1.44(1.10, 1.90)**
Richer		2.80(2.16, 3.62)***		1.67(1.19, 2.34)**
**Media exposure**				
None		Ref		Ref
Infrequent		1.06(0.86, 1.29)		1.04(0.85, 1.28)

Frequent		1.37(1.12, 1.68)**		1.31(1.07, 1.61)**
**Community-level factors**				
**Residence**				
Rural			Ref	Ref
Urban			1.60(1.19, 2.16)**	1.02(0.73, 1.43)
**Region**				
Large central			Ref	Ref
Small peripheral			0.82(0.65, 1.03)	1.04(0.81, 1.32)
Metropolitan			1.61(1.22, 2.13)**	1.74(1.29, 2.34)***
**Health facility distance**				
Big problem			Ref	Ref
Not big problem			1.06(0.91, 1.23)	0.97(0.84, 1.14)
**Community literacy**				
Low			Ref	Ref
Moderate			1.18(0.86, 1.62)	1.04(0.76, 1.41)
High			2.05(1.48, 2.85)***	1.51(1.07, 2.12)*
**Community poverty**				
Low			Ref	Ref
Moderate			0.80(0.59, 1.07)	0.94(0.70, 1.26)
High			0.73(0.53, 1.01)	0.90(0.64, 1.25)
Very high			0.54(0.39, 0.76)***	0.69(0.48, 0.99)*
Random effect				
Community variance (SE)	1.07(0.131)	0.52(0.081)	0.84(0.159)	0.50(0.080)
ICC (%)	24.49	13.71	14.66	13.28
PCV (%)	Ref	51.40	21.49	53.27
MODEL FITNESS				
Log likelihood	-2991.29	-2836.93	-2864.25	-2814.66
AIC	5986.58	5717.85	5750.50	5691.32
BIC	5999.44	5859.22	5821.19	5890.52

*significant at p<0.05; **significant at p <0.01; ***significant at p<0.001

AOR (95%CI): Adjusted Odds Ratio at 95% confidence level

AIC: Akaike information criterion

BIC: Bayesian information criterion

PCV: proportion change in variance

ICC: Intraclass correlation
